# Psychometric testing of the training needs for advance care planning scale for clinicians and nurses

**DOI:** 10.1186/s12912-024-01952-7

**Published:** 2024-07-15

**Authors:** Chunju He, Tiaoxia Dong, Jin Tan, Liu Yang, Yeyin Qiu, Jianghui Zhang, Yi Huang, Aiting Zhou, Xianlin Wang, Yuan Huang, Minglan Zhu, Simon Ching LAM, Renli Deng

**Affiliations:** 1 Department of Nursing, Affiliated Hospital of Zunyi Medical University, Guizhou Province, China; 2 Affiliated Hospital of Zunyi Medical University, Guizhou Province, China; 3 The Fifth Affiliated Hospital of Zunyi Medical University, Guangdong Province, China; 4 Tung Wah College HK, Hong Kong, China

**Keywords:** Advance care planning, Questionnaire, Reliability, Validity, Chinese clinicians and nurses

## Abstract

**Background:**

Studies have shown that Chinese Clinicians and nurses have positive attitudes toward ACP, but no local tools exist to assess their need for ACP knowledge and skills training. resulting in their inability to initiate ACP conversations as well as poor end-of-life care for patients. Therefore, this study aims to assess the needs of Chinese Clinicians and nurses for ACP knowledge and skills training and assess the validity and reliability of a questionnaire on the Training Needs for Advance Care Planning (TNACP) scale.

**Methods:**

From October to November 2021, 170 clinicians and nurses were pre-surveyed using a preliminary draft of the questionnaire. The responses were screened using item analysis, Cronbach’s alpha coefficient, and the intraclass correlation coefficient (ICC) to describe the internal consistency and stability of the questionnaire. The Content validity index (CVI), Exploratory factor analysis (EFA) and Confirmatory factor analysis (CFA) were used to test the validity of the questionnaire.

**Results:**

After independent samples t-test analysis, Except for the entry “A2”, the critical ratio between the two groups of the remaining 23 items was statistically significant (*p* < 0.05). Based on the above screening methods, the “A2” item was deleted, and the final number of questionnaire items was 23. The I-CVI was 0.79–1.00, and the S-CVI/Ave was 0.90. Three common factors were extracted—the cumulative contribution rate was 69.969%, and the factor loading of all items was 0.506–0.843 (all > 0.40). The results of confirmatory factor analysis showed that the Training Needs for Advance Care Planning (TNACP) scale model fit well(X^2^/*df* = 2.504, RMSEA = 0.092, GFI = 0.809, AGFI = 0.745, CFI = 0.931, IFI = 0.932, TLI = 0.916); the Cronbach’s α = 0.888 for the total questionnaire, and the three dimensions of Cronbach’s α were 0.729 to 0.959; and the ICC for the overall scores between the test-retest evaluations was 0.884 (*p* < 0.001).

**Conclusions:**

The TNACP scale has good reliability and validity and can be used to assess Chinese Clinicians and nurses’ training needs for implementing ACP.

**Supplementary Information:**

The online version contains supplementary material available at 10.1186/s12912-024-01952-7.

## Background

Advance care planning (ACP), a new “patient-centered” intervention that fully respects human rights, is a communication process for adults of any age and health stage that helps Clinicians and nurses to understand patients’ values, life goals, and future healthcare preferences [1, 2]. There is growing evidence that the use of ACP can facilitate communication between patients and families and Clinicians and nurses, avoid overmedication, respect patient autonomy, improve the quality of end-of-life care, and reduce healthcare costs [3–6]. Despite these benefits, there are many barriers to implementing the ACP process, including a lack of confidence among Clinicians and nurses, which is related to their lack of knowledge of and skills for delivering ACP [7–9]. Clinicians and nurses are the main force in ACP practice, playing multiple roles as assessors, educators, implementers, and coordinators in ACP practice. The process of ACP practice by Clinicians and nurses is affected by many of their own factors, such as the experience of Clinicians and nurses in facing and/or dealing with death, whether they have received professional ACP training, and their own ACP qualities [10]. Studies have shown that the inability of Clinicians and nurses to initiate ACP conversations with patients to obtain relevant information about end-of-life care and to make decisions about their care plans leads to poor quality of dying [8, 11]. The results of a series of cross-sectional studies showed that Chinese Clinicians and nurses had positive attitudes toward ACP, they agreed with its usefulness, and they were willing to promote it [12–15], but it was difficult to apply it in clinical practice due to insufficient knowledge and lack of relevant skills training, which was also consistent with the results of the study by Shepherd et al [16].

Ersek et al. [17] noted that in any clinical setting, the assessment of participants’ learning needs can effectively drive the implementation of educational programs. Therefore, to enhance the effectiveness of Clinicians and nurses’ training on ACP knowledge and skills, it is necessary to understand their training needs and provide them with targeted ACP training. Scholars in other countries have conducted a series of studies on the direct stakeholders of ACP (patients, family members, and Clinicians and nurses), and the research content is relatively comprehensive: from the investigation of the factors influencing ACP for different groups, the implementation of the intervention program, to the development of communication strategies and the testing of the effects of a series of practice studies [18]. However, research on ACP in mainland China is still in the exploratory stage, and research on ACP for different stakeholders is in an uneven state: for patients with different terminal illnesses and their families, domestic scholars have carried out studies on the acceptance and influencing factors of ACP, communication modes (tools), and empirical evidence of interventions [19].Currently, there have been domestic studies on ACP training for Clinicians and nurses [20], but a literature search did not reveal any domestic studies involving the assessment of training needs for ACP for Clinicians and nurses. Therefore, using the performance analysis model as a guiding framework, The performance analysis model is a model that has been naturally developed and summarized in the practice of training needs analysis, and its basic concept is that not all performance gaps can be transformed into training needs, and only if the performance gaps are due to a lack of knowledge and skills, and not due to other factors (e.g., inappropriate rewards and penalties, insufficient feedback, and insufficient resources) that have an impact on the work behaviors of the employees, the analysts can consider them to be training needs [21]. We initially constructed a library of entries for the ACP training needs assessment tool through literature analysis and data review in the first phase, and then translated and Chineseized the initially constructed English entries based on the Brislin translation model and revised the entries through Delphi expert consultation and pre-survey. The process of developing and compiling this assessment tool has resulted in a Chinese-language article and a successful journal publication in 2022 [22]. The purpose of this study was to validate the reliability and validity of this advance care planning training needs (TNACP) scale on this basis in order to understand the training needs of Chinese healthcare workers in terms of ACP knowledge and skills and to develop an individualized ACP training program for them. Promote a positive change in the attitude of Clinicians and nurses toward ACP, enhance their knowledge of ACP, and help promote the work of ACP in clinical practice.

## Methods

### Design

This study utilized purposive sampling through a cross-sectional survey to assess the current state of demand for the TNACP scale and to test its psychometric properties. Scale testing consists of two phases: item analysis and reliability and validity testing.

### Setting and participants

This study was conducted from October to November 2021 at the First Affiliated Hospital and Second Affiliated Hospital of Zunyi Medical University, Guizhou Province, China. The inclusion criteria for the distribution of the TNACP scale were: (1) clinical frontline doctors and registered nurses; (2) distribution in oncology, intensive care unit, respiratory and critical care medicine, cardiology intensive care unit, internal medicine-neurology, hematology, and nephrology departments; and (3) those who consented to voluntarily participate in this study. The exclusion criteria were: (1) Clinicians and nurses outside the above departments; and (2) non-clinical frontline Clinicians and nurses.

### Sample size

According to the rule of thumb, the sample size should be five to 10 times the questionnaire items, and the number of exploratory factor analysis (EFA) subjects should be at least 100 [23, 24]. Therefore, in this study, the item-to-sample ratio was 1:5. In consideration of the actual clinical situation, the final number of samples was increased by 20% to 170 to account for the rate of loss to follow-up. And 180 copies of the research questionnaire for the development of the TNACP scale (prediction version) were sent again on July 7-July 8, 2023 to conduct validation factor analysis and test the questionnaire fit.

### Measurement

This study used two self-developed questionnaires. The general information questionnaire was designed based on a literature review and clinical experience, and it included three parts: (1) a short demographic questionnaire; (2) a survey of the current situation of ACP; and (3) Clinicians and nurses’ training needs for implementing ACP. The research questionnaire for the development of the TNACP scale (prediction version) included 24 items across two dimensions (See Appendix 1).

### Phase 1: item analysis

In this study, item analysis methods, namely, correlation coefficient analysis and the critical ratio (CR) method, were used to screen the questionnaire items. The Pearson correlation coefficient method of total item scores was used to test and exclude entries with correlation coefficients < 0.40 [25]. The CR method was used to test whether the questionnaire items could identify the degree of reflection of different respondents, excluding items with a CR < 3.00 or not reaching a significant level (*p* < 0.05) [26].

### Phase 2: validity and reliability analysis

After screening the items using the above analysis methods, a formal questionnaire containing two dimensions and 23 items was developed to test the validity and reliability of the TNACP scale.

### Validity

To test the questionnaire’s validity, content validity and construct validity were assessed. The content validity index (CVI) was used to describe the content validity of the questionnaire. Experts evaluated the relevance of the questionnaire items and their corresponding dimensions on a 4-point Likert scale, from 1 = “very irrelevant” to 4 = “very relevant.” the calculated content included the item-level CVI (I-CVI) of each item in the questionnaire and the scale-level CVI (S-CVI/Ave) of the entire questionnaire. It is generally recommended that the I-CVI should be ≥ 0.78 and the S-CVI/Ave ≥ 0.80 to reflect the effectiveness of the content [27].

Construct validity was evaluated by EFA. First, the applicability of factor analysis was determined by evaluating Kaise-Meyer-Olkin’s (KMO) measure of sampling adequacy and Bartlett’s test of sphericity [28]. Bartlett’s test of sphericity reached statistical significance, and a KMO value of > 0.60 was considered suitable for factor analysis [24]. Then, the principal component analysis method and the varimax (variation maximization) rotation method were used for factor analysis [29]. The basis for extracting common factors was as follows: after orthogonal rotation by the maximum variance method, the comprehensive eigenvalue > 1.00 was extracted, the variance contribution degree exceeded 60%, and the inflection point of the scree plots was comprehensively judged [30].

### Reliability

The Cronbach’s α coefficient evaluated the internal consistency of the questionnaire. It is generally believed that if Cronbach’s α ≥ 0.70 [31], then the consistency of the questionnaire is good. To calculate the intraclass correlation coefficient (ICC), a test-retest evaluation was conducted with 16 eligible Clinicians and nurses during a two-week interval to ensure the stability of the analysis. The ICC was interpreted according to Koo and Li [32], in which values < 0.50 indicate poor reliability, values between ≥ 0.50 and < 0.75 indicate moderate reliability, values between ≥ 0.75 and < 0.90 indicate good reliability, and values ≥ 0.90 indicate excellent reliability.

### Data collection

To facilitate the study participants in filling out the questionnaire, protecting their privacy, and taking into account the needs of local “epidemic prevention and control,” this study adopted an electronic questionnaire, which was published on “Questionnaire Star” (http://www.wjx.cn), to generate online data collection. The questionnaire was available from the date of release until the sample size was met, the respondents submitted their questionnaire after completing all the required answers, and the data were uploaded to the background.

### Data entry and statistical analysis

Excel (version 2019; Microsoft, Redmond, WA, USA) was used for data organization, SPSS (version 18.0; SPSS Inc., Chicago, IL, USA) statistical software was used for statistical analysis, and AMOS programs were used for confirmatory factor analysis to calculate the overall model adaptation indicators, The main parameters are as follows: The ratio of Chi-square degrees of freedom (X2/df), root mean square error of approximation (RMSEA), goodness-of-fit index, GFI), adjusted goodness of fit index (AGFI), comparative fit index (CFI), incremental fit index (incremental fit index) IFI) and the Tacker-Lewis index (TLI). A database review system was established to ensure accurate data entry.

### Ethical consideration

This study was approved by the Ethics Review Committee of the Affiliated Hospital of Zunyi Medical University under the approval number KLLY-2021-073, with an approval date of September 1, 2021. All participants received written informed consent.

## Results

The demographic characteristics of the participants are shown in Table 1. Of the 170 questionnaires that were distributed, 159 were returned for a return rate of 93.5%. Of all the participants, 71.1% were female, 94.3% had no religious affiliation, more than half were between the ages of 26 and 35, most had a bachelor’s degree, and clinical nurses accounted for 79.2% of the total number of participants.

**Table 1:** Demographic characteristics of the participants (*N* = 159)

Demographic Characteristics		Number of Participants (*n*)	Percentage (%)
Gender	Male	46	28.9
	Female	113	71.1
Nationality	Han	138	86.8
	Minority	21	13.2
Religious beliefs	None	150	94.3
	Buddhism	8	5.1
	Taoism	1	0.6
Age	≤ 25	12	7.5
	26 to 35	95	60
	36 to 45	40	25
	≥ 46	12	7.5
Working years	1 year or less	10	6.3
	1 to 5 years	26	16.3
	6 to 10 years	41	25.8
	11 to 15 years	48	30.2
	16 to 20 years	16	10.1
	≥ 21 years	18	11.3
Education	Post-secondary degree	10	6.3
	Bachelor’s degree	135	84.9
	Master’s degree or above	14	8.8
Position	Doctor manager	4	2.5
	Clinician	24	15.1
	Nurse manager	5	3.2
	Clinical nurse	126	79.2
Title	Primary	79	49.7
	Intermediate	60	37.7
	Senior	20	12.6
Department	Respiratory Medicine	28	17.6
	Oncology	21	13.2
	Nephrology	24	15.1
	Cardiology Intensive Care Unit	22	13.8
	Internal Medicine-Neurology	24	15.1
	Hematology	20	12.6
	Intensive Care Unit	20	12.6

During in the second round of questionnaire distribution, a total of 180 questionnaires were returned. Of all participants, 83.8% were female, 72.7% had a bachelor’s degree or higher, 72.2% were between 30 and 49 years of age, and nurses accounted for 73.3% of the total number of participants, with specific demographic characteristics shown in Table 2. Data from this round of surveys were used for validation factor analysis.

**Table 2:** Demographic characteristics of the participants (*N* = 180)

Demographic Characteristics		Number of Participants (*n*)	Percentage (%)
Gender	Male	29	16.2
	Female	151	83.8
Age	22 to 29	36	19.8
	30 to 39	89	49.4
	40 to 49	41	22.7
	50 to 59	13	7.2
	≥ 60	1	0.05
Working years	1 year or less	8	4.4
	1 to 5 years	28	15.5
	6 to 10 years	42	23.3
	11 to 15 years	54	30.0
	16 to 20 years	15	8.3
	≥ 21 years	33	18.3
Education	Post-secondary degree	49	27.2
	Bachelor’s degree	123	68.3
	Master’s degree or above	8	4.4
Position	Doctor manager	12	6.6
	Clinician	36	20
	Nurse manager	6	3.3
	Clinical nurse	126	70.0
Title	Primary	82	45.5
	Intermediate	75	41.6
	Senior	23	12.7

### Item analysis

Pearson correlation analysis was used to calculate the correlation coefficient between each item and the total score of the questionnaire, the total score of each item and dimension, and the total score of each dimension and the total score of the questionnaire. The correlation coefficient for “A2” was<0.40, so the item was removed.

The questionnaires were divided into two groups, with the highest 25 to 33% of scores representing the high group and the lowest 25 to 33% representing the low group. The CR values of the 23 items in the two groups, excluding “A2,” were statistically significant (*p* < 0.05) according to the independent samples t-test analysis, ranging from 4.372 to 21.663. as shown in Table 3.

**Table 3:** Critical ratio analysis of each item

Entry	t	*P*
A1	5.074	<0.001^*^
A2	-0.650	0.522
A3	15.569	<0.001^*^
A4	-4.983	<0.001^*^
A5	8.345	<0.001^*^
A6	-11.000	<0.001^*^
A7	4.372	<0.001^*^
B1	10.590	<0.001^*^
B2	13.220	<0.001^*^
B3	10.590	<0.001^*^
B4	10.165	<0.001^*^
B5	11.349	<0.001^*^
B6	5.339	<0.001^*^
B7	10.161	<0.001^*^
C1	17.694	<0.001^*^
C2	17.694	<0.001^*^
C3	16.615	<0.001^*^
C4	11.000	<0.001^*^
C5	21.663	<0.001^*^
C6	19.887	<0.001^*^
C7	12.315	<0.001^*^
C8	13.274	<0.001^*^
C9	8.901	<0.001^*^
C10	9.046	<0.001^*^

Note: * The difference between the two groups is statistically significant

Based on the results of the correlation coefficient analysis and CR method, after comprehensive consideration and discussion, the research team decided to delete item “A2.” The final questionnaire covers 23 entries, see Appendix 2.

### Validity

#### Content validity

The content validity of the questionnaire was assessed by 14 experts. All of these experts had associate or higher titles, bachelor or higher degrees, and 50% of them had full senior titles. They had been working in relevant fields for years, ranging from five to 35 years, with clinical work and research experience. According to the formula, the I-CVI value of each item was 0.79 to 1.00 and the S-CVI/Ave was 0.90, indicating satisfactory content validity of the questionnaire.

### Construct validity

The statistical significance of Bartlett’s sphericity test was significant (χ2= 3209.318; *p* ≤ 0.001), and a KMO value of 0.945 (>0.60) was obtained. In this study, three common factors were extracted from the 23 items using principal component analysis, with a cumulative contribution of 69.969%: Factor 1 “ACP communication skills” (10 items, explained variance = 36.364); Factor 2 “ACP communication, specialist knowledge” (seven items, explained variance = 19.342); and Factor 3 “ACP communication, basic knowledge” (six items, explained variance = 14.263).The factor loadings of the 23 items in the questionnaire ranged from 0.506 to 0.843, which met the criteria for item retention (factor loadings of items > 0.40) [33], as detailed in Table 4, indicating that the public factor explained each item better:

**Table 4:** Factor loading matrix (normalized varimax rotation method)

Dimension	Entry	Factor 1	Factor 2	Factor 3
	C1	0.750		
	C2	0.804		
	C3	0.826		
	C4	0.820		
ACP communication skills	C5	0.786		
	C6	0.720		
	C7	0.724		
	C8	0.821		
	C9	0.764		
	C10	0.748		
	B1		0.804	
	B2		0.803	
	B3		0.637	
ACP communication, specialist knowledge	B4		0.664	
	B5		0.690	
	B6		-0.569	
	B7		0.556	
	A1			-0.843
	A3			0.796
	A4			-0.697
ACP communication, basic knowledge	A5			-0.626
	A6			0.697
	A7			0.506

**Fig. 1:**
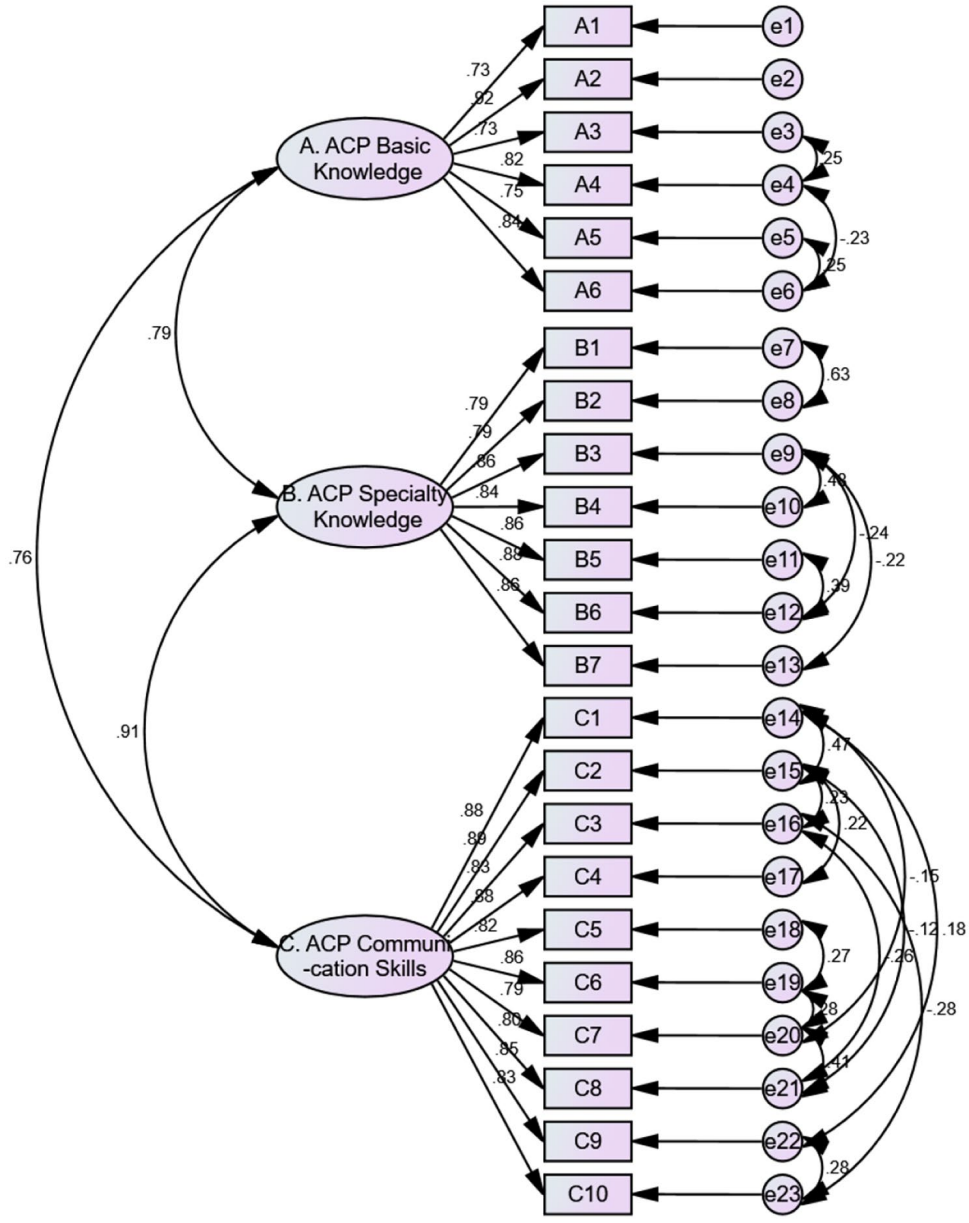
The Training Needs for Advance Care Planning (TNACP) scale Confirmatory factor analysis structural model (*n* = 180)

The results of confirmatory factor analysis show (see Fig. 1) that the overall fitting of the model is satisfactory, and the model fitting index is as follows [34]: (X^2^/*df* = 2.504, RMSEA = 0.092, GFI = 0.809, AGFI = 0.745, CFI = 0.931, IFI = 0.932, TLI = 0.916).

### Reliability

The overall Cronbach’s α coefficient for the questionnaire was 0.888 and the three-dimension Cronbach’s α coefficients ranged from 0.729 to 0.959.The ICC value for the retest reliability was 0.884 (*p* < 0.001), indicating good retest reliability. Table 5 summarizes the validation of the TNACP scale:

**Table 5:** Summary of the validation of the TNACP scale

	Methods	Statistical Methods	Results
**Item analysis**			
Item selection	Correlation coefficient analysis. critical ratio	Pearson correlation coefficient. critical ratio	Except for “A2,” the correlation coefficient of the 23 items > 0.40; except for “A2,” the CR value of each item was between 4.372 and 21.663
**Reliability**			
1. Internal consistency	Cronbach’s α	Cronbach’s α	Total Cronbach’s α = 0.888. three-dimension Cronbach’s α = 0.729 to 0.959
2. Stability	Two-week test-retest reliability	Intraclass correlation coefficient	ICC = 0.884, *p*<0.001 95%CI = 0.857–0.909
**Validity**			
1. Content validity	Reviewed by expert panel (*n* = 14)	Content validity index	I-CVI = 0.79 to 1.00. S-CVI = 0.90
2. Construct validity	Factor analysis	Exploratory factor analysis	KMO = 0.945. Bartlett’s test of sphericity: chi-squared = 3209.318. *p* ≤ 0.001. total variance explained = 69.969%
		Confirmatory factor analysis	(X2/df = 2.504, RMSEA = 0.092, GFI = 0.809, AGFI = 0.745, CFI = 0.931, IFI = 0.932, TLI = 0.916).

Note: CR = critical ratio; ICC = intraclass correlation coefficient; CI = confidence interval; I-CVI = item-level content validity index; S-CVI = scale-level content validity index; KMO = Kaise-Meyer-Olkin (measure of sampling adequacy)

## Discussion

### Availability of the TNACP scale

In contrast to the serious impediments that have slowed the development of ACP in China [20, 35], including the lack of ACP training for healthcare workers in China and superficial and insignificant training effects, the TNACP scale addresses a moderate number of questions, contains branching logic, and is usable for the intended population. The questionnaire was developed under the guidance of the performance analysis model derived from the training needs analysis theory. It provides standardized guidance for the items included in the later questionnaire, so that the overall layout of the questionnaire is closely linked with the research content. A small sample pre-survey was conducted to revise the semantics of the entries to ensure that the included English entries conformed to the conventions of Chinese expression in terms of content expression. As well as the correlation coefficient analysis method, critical ratio analysis method to ensure that the questionnaire and the dimensions, dimensions and entries, and entries and the rationality of the layout of the entries, this study in the entries after further screening to remove the “entry A2”, analyze the reasons for the content of A2 is the progress of the ACP law, the ACP law in China is still in its infancy [36], taking into account that this study is mainly to investigate the ACP knowledge of health care workers, its knowledge is the connotation and concept of ACP, ACP progress has not been investigated and listed in the group after comprehensive consideration and discussion. After the comprehensive consideration of the current study is to investigate the ACP knowledge of health care workers, its knowledge is the connotation and concept of ACP, ACP progress is not investigated and listed, in the comprehensive consideration and discussion of the group, the decision was made to delete the entry; the reliability and validity analysis of the questionnaire from an objective point of view again adjusted and confirmed the scientific rationality of the questionnaire’s overall structure and content. And using literature analysis as the research method, the validity of the content was ensured through entry screening and expert evaluation, and the usability of the tool was further demonstrated through the principal component analysis method, EFA, and reliability verification.

### Reliability and validity

The TNACP scale is considered a reliable and valid tool for assessing Chinese Clinicians and nurses’ needs in obtaining ACP knowledge and skills.

#### Validity

Questionnaire validity is used as an indicator to specifically examine the energy efficiency of each item, i.e., whether each item plays an important role for the questionnaire as a whole [37]. The test validity of this study includes structural validity and content validity, and the methods used for structural validity test are exploratory factor analysis (EFA) and confirmatory factor analysis (CFA), because the dimensions of this study’s self-administered questionnaire are unknown compared with the mature scale. Firstly, the EFA is used to find out the number of factors affecting the observed variables and the degree of correlation between each factor and each observed variable, and secondly, the CFA is used to verify the ability of the model with the factors defined beforehand to fit the actual data, and the final test results come out that the structure is consistent with the observed data; content validity is reflected by the content validity index. The results of the exploratory factor analysis showed that there were three common factors extracted, which were different from the number of dimensions that were originally classified in this study based on the theory of training needs alone. Based on the analysis of the results, it was found that the three common factors were related to ACP basic knowledge, ACP specialty knowledge, and ACP communication skills, so this study reclassified the three dimensions in the follow-up study and refined the knowledge dimensions into two dimensions: basic and specialty knowledge. The content validity index (I-CVI) of each item of the questionnaire in this study ranged from 0.79 to 1.00, which was greater than 0.78, indicating good content validity. Comprehensively analyzing the results of structural and content validity of the questionnaire, it can be concluded that each item plays an important role in the whole questionnaire, and the dimensional division of the questionnaire after structural validity analysis is more scientific and reasonable.

### Reliability

The questionnaire had satisfactory internal consistency, with a total Cronbach’s α = 0.888 and Cronbach’s α > 0.70 for all dimensions. Although the ICC was good in retest reliability, it did not reach a very high satisfactory level; it was speculated that this was due to the shallow and simple knowledge and mastery of ACP among the participants and the passage of time with busy clinical work. However, the ICC = 0.884, which indicated that the questionnaire’s retest reliability was generally satisfactory.

#### Practical significance of the questionnaire

Currently, there are few studies on ACP training needs for healthcare workers and there is no established survey tool for scholars in the related fields to use. Based on the existing international research on the knowledge and communication skills that healthcare workers should have in order to implement ACP, this study developed an ACP training needs survey tool for healthcare workers in China, which can provide a reference value and a research basis for scholars in China to carry out relevant research. ACP training for healthcare workers is still in its infancy in China, and this status quo is an important factor leading to the slow development of ACP in China, so it is very necessary to carry out the relevant pre-training needs survey, and the relevant survey tool is also very practical and practical value and guiding significance.

## Conclusions

The TNACP scale constructed in this study had three dimensions and 23 items and was found to be comprehensive, scientific, reliable, and valid. This scale can be used to assess the training needs of Chinese Clinicians and nurses in implementing ACP, address the “knowledge gap” among Clinicians and nurses, and provide a targeted reference for improving their knowledge of and skills for the delivery of ACP.

### Limitations

The sample for this study was drawn from two tertiary care hospitals with a relatively homogeneous population. In the future, further exploration and analysis will be conducted by including Clinicians and nurses from community hospitals and nursing homes. Another limitation of this study was the small sample size analyzed, but it also provided useful information. In the future, validated factor analysis will be used in a larger sample for further evaluation.

### Electronic supplementary material

Below is the link to the electronic supplementary material.

Supplementary Material 1

Supplementary Material 2

## Data Availability

The datasets used and/or analyzed during the current study are available from the corresponding author on reasonable request.

## Abbreviations

ACP: Advance care planning
CR: Critical ratio
CVI: Content validity index
EFA: Exploratory factor analysis
ICC: Intraclass correlation coefficient
I-CVI: Item-level content validity index
KMO: Kaise-Meyer-Olkin (measure of sampling adequacy)
S-CVI/Ave: Scale-level content validity index/average
TNACP: Training Needs for Advance Care Planning
