# Supplementary material for: Psychometric testing of the training needs for advance care planning scale for clinicians and nurses
Source: BMC Nurs. 2024 Jul 15;23:476. doi: 10.1186/s12912-024-01952-7 (PMC11247906; doi:10.1186/s12912-024-01952-7)
Supplement: Supplementary file 1 — Supplementary Material 1 [file 12912_2024_1952_MOESM1_ESM.docx]

**Appendix 1：**The Training Needs for Advance Care Planning (TNACP) scale for Chinese healthcare providers

| Serial number | Entry content | | | | Expert opinion | | | | | |
| --- | --- | --- | --- | --- | --- | --- | --- | --- | --- | --- |
| **Content I: ACP knowledge** | | | | | | | | | | |
| **A1** | Correct | Error | | Not sure | Very important | More important | Important | Not too important | Not important | Modification Comments |
| Medical autonomy refers to the patient's right to make independent, self-directed decisions in medical activities, and this autonomy expresses fundamentally the patient's right to choose the course of his or her own life. | | | | | □ | □ | □ | □ | □ |  |
| **A2** | Correct | Error | | Not sure | Very important | More important | Important | Not too important | Not important | Modification Comments |
| Currently ACP has been legislated and implemented in Hong Kong, Taiwan and some inland areas of China. | | | | | □ | □ | □ | □ | □ |  |
| **A3** | Correct | Error | | Not sure | Very important | More important | Important | Not too important | Not important | Modification Comments |
| ACP is a process in which patients discuss their future treatment and care wishes with their health care provider, family, and friends, before they may lack or lose the ability to express their treatment wishes. | | | | | □ | □ | □ | □ | □ |  |
| **A4** | Correct | Error | | Not sure | Very important | More important | Important | Not too important | Not important | Modification Comments |
| ACP is available to, but not limited to, adults with medical decision-making capacity. ACP can also be administered to minors and adults without decision-making capacity, but the implementation of their ACP is individualized depending on the circumstances; the core principle is to inform patients truthfully about their condition. | | | | | □ | □ | □ | □ | □ |  |
| **A5** | Correct | Error | | Not sure | Very important | More important | Important | Not too important | Not important | Modification Comments |
| An advance directive (AD), also known as an advance directive, is a verbal or written agreement that can be set up as a proxy and updated according to the patient's wishes, for medical care in the event that the individual is conscious and has the ability to make decisions and is unable to make judgments about his or her own condition (respiratory, cardiac arrest, etc.). | | | | | □ | □ | □ | □ | □ |  |
| **A6** | Correct | Error | | Not sure | Very important | More important | Important | Not too important | Not important | Modification Comments |
| Substitute decision maker (SDM), also known as a durable power of attorney, is a person who is appointed to make financial decisions on behalf of a patient when he or she is too sick to make those choices on his or her own. | | | | | □ | □ | □ | □ | □ |  |
| **A7** | Correct | Error | Not sure | | Very important | More important | Important | Not too important | Not important | Modification Comments |
| The ACP was created to facilitate the signing of the AD; the difference is that the ACP is essentially a communication process and the AD is a legal instrument. the implementation of the ACP facilitates the establishment of the SDM; in distinction the ACP is a multi-party communication process and the SDM is an individual who makes decisions instead of the patient within the scope of his or her authorization. | | | | | □ | □ | □ | □ | □ |  |
| **B1** | Correct | Error | Not sure | | Very important | More important | Important | Not too important | Not important | Modification Comments |
| Be aware of the benefits and prognosis of in-hospital cardiopulmonary resuscitation. | | | | | □ | □ | □ | □ | □ |  |
| **B2** | Correct | Error | Not sure | | Very important | More important | Important | Not too important | Not important | Modification Comments |
| Know the prognosis of common chronic diseases (e.g., hypertension, diabetes). | | | | | □ | □ | □ | □ | □ |  |
| **B3** | Correct | Error | Not sure | | Very important | More important | Important | Not too important | Not important | Modification Comments |
| Be aware of the prognosis of common acute and critical symptoms in the ICU. | | | | | □ | □ | □ | □ | □ |  |
| **B4** | Correct | Error | Not sure | | Very important | More important | Important | Not too important | Not important | Modification Comments |
| Clear control of late life series symptoms (pain, dyspnea, delirium and agitation). | | | | | □ | □ | □ | □ | □ |  |
| **B5** | Correct | Error | Not sure | | Very important | More important | Important | Not too important | Not important | Modification Comments |
| Be able to clarify the patient's social relations and relatives' opinions. | | | | | □ | □ | □ | □ | □ |  |
| **B6** | Correct | Error | Not sure | | Very important | More important | Important | Not too important | Not important | Modification Comments |
| Able to develop a variety of comprehensive treatment and care plans, including active and palliative care, based on the patient's condition and ethical principles. | | | | | □ | □ | □ | □ | □ |  |
| **B7** | Correct | Error | Not sure | | Very important | More important | Important | Not too important | Not important | Modification Comments |
| The ability to ensure that different forms of therapeutic care options are presented to the patient without personal bias. | | | | | □ | □ | □ | □ | □ |  |
| **Content Two: ACP Communication** | | | | | | | | | | |
| **C1** | Correct | Error | Not sure | | Very important | More important | Important | Not too important | Not important | Modification Comments |
| Ability to correctly assess the patient's ability to discuss ACP. | | | | | □ | □ | □ | □ | □ |  |
| **C2** | Correct | Error | Not sure | | Very important | More important | Important | Not too important | Not important | Modification Comments |
| Clearly prepare people and environment before ACP implementation. | | | | | □ | □ | □ | □ | □ |  |
| **C3** | Correct | Error | Not sure | | Very important | More important | Important | Not too important | Not important | Modification Comments |
| Be clear about when and with whom to discuss the ACP. | | | | | □ | □ | □ | □ | □ |  |
| **C4** | Correct | Error | Not sure | | Very important | More important | Important | Not too important | Not important | Modification Comments |
| Ability to use structured and flexible communication skills (e.g., alternating short and complex responses, silence, repetitive discussions, asking about the future with "what ifs", citing one's own example, respecting the patient, and expressing one's wishes and concerns as a medical professional). | | | | | □ | □ | □ | □ | □ |  |
| **C5** | Correct | Error | Not sure | | Very important | More important | Important | Not too important | Not important | Modification Comments |
| It simplifies communication and avoids redundant words. | | | | | □ | □ | □ | □ | □ |  |
| **C6** | Correct | Error | Not sure | | Very important | More important | Important | Not too important | Not important | Modification Comments |
| Be able to identify the needs of patients from listening. | | | | | □ | □ | □ | □ | □ |  |
| **C7** | Correct | Error | Not sure | | Very important | More important | Important | Not too important | Not important | Modification Comments |
| The ability to avoid interrupting and influencing the patient's decision making. | | | | | □ | □ | □ | □ | □ |  |
| **C8** | Correct | Error | Not sure | | Very important | More important | Important | Not too important | Not important | Modification Comments |
| Ability to conduct verbal handoffs with colleagues and multidisciplinary teams using a structured format (e.g. ISBAR). | | | | | □ | □ | □ | □ | □ |  |
| **C9** | Correct | Error | Not sure | | Very important | More important | Important | Not too important | Not important | Modification Comments |
| Ability to implement post AD and SDM tracking. | | | | | □ | □ | □ | □ | □ |  |
| **C9** | Correct | Error | Not sure | | Very important | More important | Important | Not too important | Not important | Modification Comments |
| Ability to handle tri-partite decision making conflicts between doctors, patients and families. | | | | | □ | □ | □ | □ | □ |  |
