# Supplementary material for: Psychometric testing of the training needs for advance care planning scale for clinicians and nurses
Source: BMC Nurs. 2024 Jul 15;23:476. doi: 10.1186/s12912-024-01952-7 (PMC11247906; doi:10.1186/s12912-024-01952-7)
Supplement: Supplementary file 2 — Supplementary Material 2 [file 12912_2024_1952_MOESM2_ESM.docx]

**Appendix 2：**The Training Needs for Advance Care Planning (TNACP) scale for Chinese healthcare providers

| Entry | | Correct | Error | | Not sure | |
| --- | --- | --- | --- | --- | --- | --- |
| 1. ACP Basic Knowledge | A1. Medical autonomy refers to the patient's right to make independent, self-directed decisions in medical activities, and this autonomy expresses fundamentally the patient's right to choose the course of his or her own life. |  |  | |  | |
|  | A2.ACP refers to a process that supports adults of any age or health stage to understand and share their personal values, life goals, and future health care preferences. |  |  | |  | |
|  | A3.ACP is applicable to both adults and minors, and the implementation of ACP for minors and adults without decision-making ability will be individualized depending on the situation; the core principle is to inform patients truthfully about their conditions. |  |  | |  | |
|  | A4. Advance directives (ADs) are documented statements of medical treatment and personal values, beliefs, and/or personally designated agents of medical choice that an individual will accept or refuse in the event that he or she loses decision-making capacity in the future, while conscious and capable of making decisions. |  |  | |  | |
|  | A5. Substitute decision maker (SDM), also known as a durable power of attorney, is a person who is appointed to make financial decisions in place of the patient when the patient is too sick to make choices on his or her own. |  |  | |  | |
|  | A6. The ACP was created to facilitate the signing of the AD; the difference is that the ACP is essentially a communication process and the AD is a legal instrument. the implementation of the ACP facilitates the establishment of the SDM; in distinction the ACP is a multi-party communication process and the SDM is an individual who makes decisions instead of the patient within the scope of his or her authorization. |  |  | |  | |
| Entry | | Strongly disagree | Disagree | Not necessarily | Agree | Strongly agree |
| 1. ACP Specialty Knowledge | B1. Be aware of the benefits and prognosis of in-hospital cardiopulmonary resuscitation. |  |  |  |  |  |
|  | B2. Be aware of the prognosis of common chronic diseases (e.g., hypertension, diabetes). |  |  |  |  |  |
|  | B3. Be aware of the prognosis of common acute and critical symptoms in the ICU. |  |  |  |  |  |
|  | B4. clear control of the late life series of symptoms (pain, dyspnea, delirium and agitation). |  |  |  |  |  |
|  | B5. Be able to clarify the patient's social relations and relatives' opinions. |  |  |  |  |  |
|  | B6. Be able to develop a variety of comprehensive treatment care plans, including active and palliative care, based on the patient's condition and ethical principles. |  |  |  |  |  |
|  | B7. be able to ensure that the different types of treatment and care options are presented to the patient without personal bias. |  |  |  |  |  |
| Entry | | Strongly disagree | Disagree | Not necessarily | Agree | Strongly agree |
| 1. ACP Communication Skills | C1. Ability to correctly assess the patient's ability to discuss ACP. |  |  |  |  |  |
|  | C2. Clearly prepare people and environment before ACP implementation. |  |  |  |  |  |
|  | C3. Be clear about when and with whom to discuss. |  |  |  |  |  |
|  | C4.Ability to use structured and flexible communication skills (e.g., alternating short and complex responses, silence, repetitive discussions, asking about the future with "what ifs", citing own examples, respecting patients, expressing wishes and concerns as a medical professional). |  |  |  |  |  |
|  | C5. The ability to simplify the content of communication and avoid complicated and redundant words. |  |  |  |  |  |
|  | C6. Be able to identify the needs of the patient from listening |  |  |  |  |  |
|  | C7. The ability to avoid interrupting and influencing the patient's decision making. |  |  |  |  |  |
|  | C8. Ability to conduct verbal handoffs with colleagues and multidisciplinary teams using a structured format (e.g. ISBAR). |  |  |  |  |  |
|  | C9. be able to implement post AD and SDM tracking. |  |  |  |  |  |
|  | C10. Be able to handle tri-partite decision making conflicts between doctors, patients and families |  |  |  |  |  |
